# FlyPhoneDB2: A computational framework for analyzing cell-cell communication in *Drosophila* scRNA-seq data integrating AlphaFold-multimer predictions

**DOI:** 10.1016/j.csbj.2025.06.032

**Published:** 2025-06-20

**Authors:** Mujeeb Qadiri, Ying Liu, Ah-Ram Kim, Myeonghoon Han, Eric Zhou, Austin Veal, Tzu-Chiao Lu, Hongjie Li, Yanhui Hu, Norbert Perrimon

**Affiliations:** a Department of Genetics, Blavatnik Institute, Harvard Medical School, Harvard University, Boston, MA 02115, USA; b Huffington Center on Aging, Baylor College of Medicine, Houston, TX 77030, USA; c Department of Molecular and Human Genetics, Baylor College of Medicine, Houston, TX 77030, USA; d Howard Hughes Medical Institute, Boston, MA 02138, USA

**Keywords:** *Drosophila*, Signal transduction, Cell-cell communication, Single cell RNA-seq

## Abstract

Cell-cell communication (CCC) plays a critical role in the physiological regulation of organisms and has been implicated in numerous diseases. Previously, we introduced FlyPhoneDB, a tool designed to explore CCC in *Drosophila* single-cell RNA-sequencing datasets. The core algorithm of FlyPhoneDB infers tissue-specific signaling events between cell types by calculating cell-cell interaction scores based on curated ligand-receptor (L-R) expression across major signaling pathways. However, the utility of FlyPhoneDB was limited by the relatively small number of available L-R pairs.

Here, we present FlyPhoneDB2, a major upgrade featuring a significantly expanded knowledgebase that includes a greater number of L-R pairs, incorporating annotations from mammalian species and structural predictions from AlphaFold-Multimer. In addition, the algorithm has been optimized for improved performance and more effective noise filtering. New functionalities have also been introduced, such as the addition of downstream reporter genes to evaluate pathway activity, multi-sample CCC comparison, and enhanced visualizations summarizing communication at a network level.

We demonstrate the utility of FlyPhoneDB2 by analyzing whole-body single-nuclei RNA-seq datasets from flies with gut tumors induced by the Yorkie oncogene. We show that FlyPhoneDB2 not only recapitulates established biological insights into the *Drosophila* Yorkie tumor model, but also identifies novel potential L-R pairs that may play important roles in tumor-induced cachexia. FlyPhoneDB2 is available at https://www.flyrnai.org/tools/fly_phone_v2/.

## 1 Introduction

Communications between different organs and cell types are crucial for maintaining the integrity and functionality of multicellular organisms, ensuring coordinated responses to internal and external changes. It occurs through several mechanisms such as direct cell-cell contact and endocrine signaling to act on distant cells. Analysis tools for studying cell-cell communication (CCC) have been advanced significantly, enabling researchers to decode complex signaling networks and interactions. These tools can be categorized based on the type of input data. For example, CellPhoneDB [1] and CellChat [2] can analyze single-cell RNA-seq (scRNAseq) data to infer CCC, while SpatialDE [3] and Giotto [4] allows to analyze spatial gene expression data for potential cell-cell interactions. These tools rely on curated databases of known ligand-receptor (L-R) pairs and use the gene expression patterns of L-R pairs from scRNAseq or spatial transcriptomic data to predict potential communication events between cell types. However, these analyses are constrained by the availability of known L-R interactions, which may restrict their applicability.

Previously, we developed FlyPhoneDB [5], a specialized database and computational tool to study CCC from scRNAseq in *Drosophila melanogaster* (fruit fly), a widely used model organism in biology. Our framework for dissecting CCC involves the co-expression of 196 L-R pairs using a manually curated L-R pair database. This database includes 196 L-R pairs, representing major signaling pathways including NOTCH signaling, JAK-STAT signaling, and many more. The log normalized expression of a known L-R pair is multiplied to produce an interaction score. To filter for cell-type specific signaling, a permutation test is carried out to reveal statistically significant candidate L-R signaling pairs for any two given cell types. FlyPhoneDB has been used in a number of studies, for example, to address CCCs between germline cells and somatic cells in the testis [6], between different cell types in Malpighian Tubules/kidney [7], between brain and body cell types during neurodegeneration [8], as well as the signaling events between different cell types in the brain of a *Drosophila* frontotemporal dementia model [9].

Here, we present FlyPhoneDB2, the latest version of FlyPhoneDB, which maintains the core algorithm of FlyPhoneDB with improved speed and additional functionality. Namely, the algorithm has been implemented as an R package with significantly improved run times. The L-R database has also been expanded to 1804 L-R pairs with varying degrees of confidence. In addition, FlyPhoneDB2 enables users to analyze pathway activity by taking into consideration downstream reporter genes alongside upstream L-R interactions. Importantly, new functionality has been implemented to investigate the differences of CCC events between multiple-samples. FlyPhoneDB2 is available as a web tool allowing researchers to upload datasets online (https://www.flyrnai.org/tools/fly_phone_v2) and as a standalone R package (https://github.com/FullStackGoogler/FlyPhoneDB2) for bioinformaticians to run analyses locally.

## 2 Materials and methods

### 2.1 Mapping of mammalian L-R pairs and curate L-R pairs

The L-R annotation for mammalian species was obtained from CellTalkDB [10]. Human/mouse genes were mapped to *Drosophila* genes using DIOPT [11] with high and moderate rank filters to exclude low confident mappings. Signal peptide predictions were performed for ligands using signal v6, while deepTMHMM [12] and TMHMM2 [13] were used to predict transmembrane (TM) proteins for receptors. Secreted protein and receptor protein annotations were obtained from UniProt and Gene Ontology. The rank, source, and mammalian orthologues of these L-R pairs are made available in the FlyPhoneDB2 knowledgebase.

### 2.2 Prediction of L-R interactions using AlphaFold-multimer

To predict interactions between L-R pairs mapped from CellTalkDB, we used the full-length protein sequences of the longest isoforms for both ligands and receptors as input to AlphaFold-Multimer. For each predicted interaction, we computed five metrics: Local Interaction Score (LIS), defined as the mean inverted-PAE over all inter-chain residue pairs with PAE ≤ 12 Å (the “local interaction area”); contact Local Interaction Score (cLIS), which restricts the same inverted-PAE calculation to residue pairs whose Cβ–Cβ distance (or Cα for glycine) is ≤ 8 Å; the product of LIS and cLIS (LIS × cLIS), capturing both interface confidence and contact specificity; interface TM-score (ipTM), the inter-chain TM-score reported by AlphaFold-Multimer and reflecting overall interface geometry accuracy; and Model Confidence, the combined pTM/ipTM score returned by AlphaFold-Multimer.

We used three large-scale yeast-two-hybrid reference datasets (yeast, *Drosophila*, and human) [14], [15], [16] together with simulated negative controls to distinguish interacting (positive) from non-interacting (negative) pairs. For each metric, we then determined a cutoff at which 10 % of negative pairs would exceed the threshold. An interaction was classified as a positive PPI if at least two of the five metrics met the 10 % FPR threshold. The analysis code for calculating LIS and cLIS is available at https://github.com/flyark/AFM-LIS.

To evaluate potential novel ligand–receptor (L–R) interactions, we performed an all-by-all prediction between selected ligands and predicted single-pass transmembrane (TM) receptors. For receptors, we selected the extracellular regions of all single-pass TM protein isoforms, as predicted by DeepTMHMM (https://dtu.biolib.com/DeepTMHMM); proteins with extracellular regions exceeding 3000 amino acids were excluded due to computational constraints. For ligands, we used the longest protein isoform with the predicted signal peptide (identified by SignalP; https://services.healthtech.dtu.dk/services/SignalP-4.1/) removed, to optimize computational efficiency. AlphaFold-Multimer predictions were conducted using LocalColabFold (v1.5.2) as described in Kim et al. [17], and downstream analyses—including calculation of mean ipTM and mean LIS—were performed according to protocols available at https://github.com/flyark/AFM-LIS.

In total, we screened 32,224 potential L–R pairs, comprising 255 receptors (424 protein isoforms) and the longest isoform for each of the 76 ligands. High-confidence manually curated L–R pairs from the original FlyPhoneDB knowledgebase were included as positive controls. Based on the performance of these positive controls, we established thresholds of mean ipTM ≥ 0.4551 or mean LIS ≥ 0.2471 to define putative interactions. Applying these cutoffs, we identified 1013 potential novel L–R pairs among the 32,224 predictions.

### 2.3 Improving the performance and visualization of FlyPhoneDB as well as developing new functionalities

FlyPhoneDB evaluates CCC events based on L-R pair annotations. For each candidate L-R pair, an interaction score is calculated as the log-normalized (log1p(x), natural log of x plus one) product of the average ligand expression in the sender cell type and the average receptor expression in the receiver cell type. The specificity and significance of the interaction score is assessed by comparing it to a null distribution of interaction scores generated by randomly shuffling cell labels 1000 times. Interactions with a p-value < 0.05 are considered statistically significant. In FlyPhoneDB, cell labels were randomly shuffled P times for each sender–receiver (i, j) pair among N cell types, leading to N^2^ × P permutations. In FlyPhoneDB2 we reduce computational complexity by generating P shuffled label assignments and reusing these fixed labels across all N^2^ sender–receiver pairs. This approach decreases the number of required calculations from N^2^ × P to P, providing a N^2^-fold speedup. For example, with N = 30 and P = 1000, this change results in a 900-fold reduction in computational time for significance testing. This approach is analogous to the random-label permutation procedure employed in Gene Set Enrichment Analysis (GSEA), which generates a fixed set of label permutations and applies these shared permutations to compute p-values across all tested gene sets [18].

While the default number of permutations remains 1000, users now have the flexibility to adjust this parameter on both the FlyPhoneDB2 web interface and standalone version. Additionally, FlyPhoneDB2 implements a new filtering step to remove low-confidence L-R pairs: interactions where ligand expression in the source cell or receptor expression in the target cell is detected in less than 10 % of cells are excluded. This enhances the robustness of the CCC results by reducing noise and improving confidence in the identified interactions.

FlyPhoneDB2 offers an updated version of the visualizations. Besides the heatmap illustration of the core-component expression for each signaling pathways, three new heatmaps summarizing the ligands, receptors and reporter genes are provided in the new version. In addition, FlyPhoneDB2 generates a dot plot in which the size and color of each dot represent the maximum expression levels of the receptor and reporter, respectively, for each signaling pathway. This integrated visualization enables the assessment of both receptor and reporter expression within target cells to facilitate analysis of pathway activity. For each signaling pathway, circle plots are provided by both the original and new FlyPhoneDB, where nodes represent cell types and the edges reflect the direction/strength of CCC by summarized interaction scores for the relevant L-R pairs. In FlyPhoneDB2, this plot has been updated to focus on only one source cell type to enhance visual clarity. On the other hand, two new types of visualizations have also been introduced: 1.) Scatter plots, which summarize the overall CCC activity per cell type. On these plots, the x-axis shows the sum of all incoming interaction scores (the sum of the CCC scores of all L-R pairs from all sender cell types), and the y-axis shows the sum of all outgoing interaction scores (the sum of CCC scores of all L-R pairs from all receiver cell types) for each cell type (sFig 2). 2.) Chord diagrams are also generated which summarize outgoing CCC from each source cell type to all target cell types. For a given cell type, the width of the bars indicates the strength of the total outgoing signals (the sum of the CCC scores of all L-R pairs as the sender cell) to various target cell types in the dataset (sFig 2 and 3).

We also introduce enhanced support for sample-level analysis through user-specified sample annotations in the metadata file. FlyPhoneDB2 automatically generates sample-specific expression matrices and performs cell–cell communication (CCC) analyses separately for each sample. For convenience and integrative analysis, FlyPhoneDB2 outputs both individual result files for each sample and a unified results file that integrates CCC scores across multiple samples. The combined file includes two types of differential CCC scores for direct comparison between experimental and control samples: (1) the log_2_ fold-change, obtained by taking the log_2_ of the score in the experimental sample divided by that in the control sample, and (2) the absolute difference, calculated by subtracting the control score from the experimental score.

Optionally, users may supply lists of differentially expressed genes (DEGs), which are then integrated into the combined results file to facilita666te identification of dysregulated signaling events. Dysregulated signaling events are defined as CCC in which the ligand and/or receptor are significantly differentially expressed across the samples. To support downstream analysis and visualization, FlyPhoneDB2 provides sample comparison visualizations that include: (i) pathway-centric heatmaps displaying differential expression among core pathway components, and (ii) differential CCC circle plots, in which nodes denote cell types, edge colors indicate the directionality (up or down regulation) of CCC changes, and edge thickness is proportional to the magnitude of the differential ligand–receptor interaction score between samples.

### 2.4 Development of the standalone package and online resource

The standalone R package (available on GitHub) was developed using R (version 4.4.0). It incorporates major libraries, such as Seurat, for storing and processing the inputted scRNAseq data. Visualizations are primarily generated using ggplot2, with additional specialized libraries like circlize, igraph, and pheatmap. The package includes both the original FlyPhoneDB knowledgebase of L-R pairs and pathway core components, as well as the updated knowledgebase from FlyPhoneDB2.

The online tool was developed as an application utilizing the Symfony framework, hosted on a traditional LAMP stack. The knowledgebase of L-R pairs and pathway core components is stored in a MySQL database. The FlyPhoneDB2 application imports the R package after obtaining and performing quality control (QC) on the data files submitted via the website. The backend was primarily developed using PHP, while the frontend views were rendered using the Twig template engine. JQuery from the JS library was used for the data browsing page, and DataTables was employed for table displays on the website. Bootstrap assisted in the creation of website elements, such as forms and other interface components. Icons throughout the application were sourced from the Font Awesome library. Both the website for the online tool and the database are hosted on the O2 high-performance computing (HPC) cluster at Harvard Medical School, maintained by the Research Computing group.

## 3 Results and discussion

**Fig. 1:**
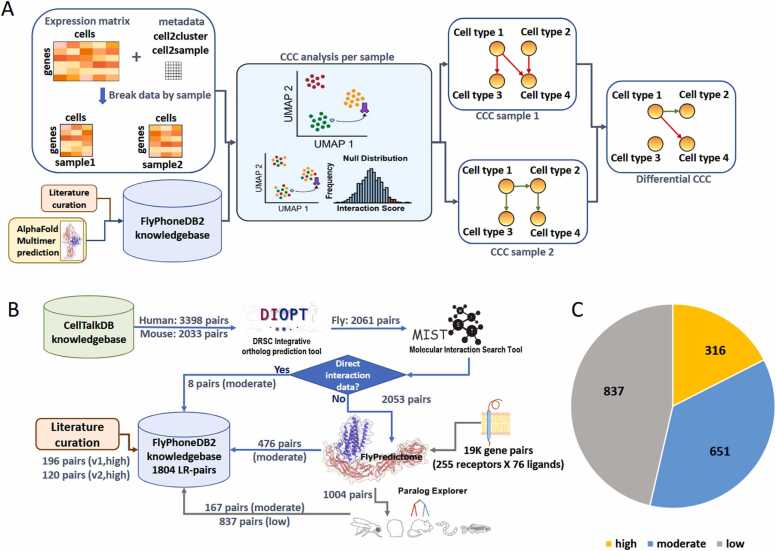
Overview of FlyPhoneDB2. A) The implementation of the new generation of FlyPhoneDB for cell-cell communication (CCC) analysis in scRNAseq datasets. The knowledge base of ligand-receptor (L-R) pairs was expanded through literature review and AlphaFold-Multimer predictions. The new pipeline allows users to specify sample annotations and generate the corresponding data matrix. Subsequently, a sample-specific CCC analysis is conducted to identify significant CCC events considering the ligand expression in the source cell and receptor expression in the target cell, followed by a permutation test. Additionally, FlyPhoneDB2 integrates the results from different samples, enabling users to compare them and identify differentially regulated CCC events. B) Workflow of knowledgebase update. C) Confidence rank for FlyPhoneDB2 knowledgebase: literature curated L-R pairs were assigned high rank, the AlphaFold-Multimer predictions with additional evidence based on orthologs and/or paralogs were assigned moderate rank while the remaining AlphaFold-Multimer predictions were assigned low rank.

In 2022, we launched FlyPhoneDB, a tool designed to study cell-cell communication (CCC) in *Drosophila* using scRNAseq data. FlyPhoneDB2 is the next generation tool with significant improvement in several key areas from database content to data analysis pipeline (Fig. 1A).

### 3.1 Expanding the knowledgebase of L-R interaction and signaling pathways

Historically, many potential L-R pairs have been genetically identified, but direct physical validation remains challenging due to the inherent complexity of membrane-bound and secreted proteins. As a result, when the first knowledgebase for L-R pair annotations in FlyPhoneDB was built using high-confidence annotations from a *Drosophila* literature review conducted by experts, its coverage was relatively limited, consisting of only 196 pairs for 96 ligands and 85 receptors, primarily from major signaling pathways. To expand the knowledgebase for *Drosophila*, we routinely incorporate high-confidence L-R pairs identified through a review of the most recent literature. This update also includes 46 hemophilic interaction pairs implicated in differential cell-cell adhesion [19], [20]. Meanwhile, the advances in artificial intelligence, most notably AlphaFold, have marked a significant leap in the field of computational biology by using deep learning to predict protein structures with remarkable accuracy. AlphaFold-Multimer extends this capability by predicting the structure of multi-protein complexes, thereby revealing how proteins interact and assemble into functional complexes. To assess the feasibility of using AlphaFold-Multimer for L-R pairs, AlphaFold-Multimer was applied on the high-confidence LR pairs curated from literature in the original FlyPhoneDB [5], using Local Interaction Score (LIS) metrics described in Kim et al. [17]. Prediction results were available for 179 of these pairs in the FlyPredictome database. Among them, 129 pairs (72.1 %) were predicted as positive interactions, confirming that literature-curated L-R pairs are generally supported by structural modeling and underscoring the utility of AlphaFold-Multimer for identifying direct ligand–receptor interactions (Sup Fig. 1).

To systematically expand the FlyPhoneDB knowledgebase and prioritize L-R pairs with higher physiological relevance, we first focused on interactions that have been identified and annotated in mammalian species. Specifically, we collected L-R pairs curated for human and mouse from CellTalkDB, the database with the highest number of annotated L-R pairs [10], and mapped the corresponding mammalian ligands and receptors to *Drosophila* orthologs using DIOPT [11]. This analysis identified 2061 fly pairs orthologous to those in human and mouse sets. However, only 8 had experimental evidence of direct interaction based on protein-protein interaction data in flies according to Molecular Interaction Search Tool (MIST) analysis [21]. Given AlphaFold-Multimer’s demonstrated ability to distinguish direct from indirect PPIs, we applied it to the remaining 2053 pairs lacking experimental validation, leading to the prediction of direct PPIs in 476 cases. These pairs were incorporated into FlyPhoneDB2 knowledgebase and assigned a moderate rank to differentiate them from high-confidence pairs curated from *Drosophila* literature. Recognizing the potential bias of literature-based curation, we next sought to identify novel interactions beyond known mammalian orthologs by systematically screening putative L-R pairs. Building upon a recent study that successfully used AlphaFold-Multimer for deorphanizing ligands to single-pass transmembrane (TM) receptors [22], we conducted an all-by-all screen of 255 single-pass TM receptors with 76 selected ligands. The source of ligand annotation (eg. UniProt), the expression level in gut tumor [23] and the protein size of ligands are considered. This screen encompassed 32,224 L-R protein-pairs, considering all protein isoforms of the 255 single-pass TM receptors (424 isoforms) and the longest protein isoform of the 76 ligands. Using ipTM and LIS analysis, we predicted 1013 direct interactions among these candidates. To further refine these predictions, we investigated cases where multiple ligands were predicted to bind to the same receptor. Since homologous ligands may share binding properties, we examined the paralog relationships using Paralog Explorer [24]. L-R pairs involving paralogous ligands were assigned a moderate rank, distinguishing them from low-confidence L-R pairs. Out of the 1013 pairs predicted for single-pass TM, the subsequent paralog analysis refined this set to 167 pairs with moderate rank, while the remaining 837 pairs were assigned low rank (Fig. 1B). Finally, we integrated the high-confidence pairs from both literature curation and AlphaFold-Multimer-based computational predictions, resulting in a comprehensive set of 1804 L-R pairs with 393 ligands and 479 receptors (Sup Table 1).

In summary, we have expanded the FlyPhoneDB knowledgebase substantially (Fig. 1B). Eighteen percent of the dataset was obtained from literature curation and assigned a high rank, while 36 % was assigned a moderate rank (Fig. 1C). The latter category included pairs identified through ortholog mapping from CellTalkDB using DIOPT and predicted by AlphaFold-Multimer as interacting pairs, as well as paralogous ligands predicted to bind the same receptor based on AlphaFold-Multimer predictions (Fig. 1B). The tool allows users to select L-R pairs based on confidence rank, with the source of each pair clearly annotated, enabling researchers to refine their results accordingly. Additionally, FlyPhoneDB2 now provides signal peptide predictions for ligands, transmembrane domain annotations and prediction for receptors, and incorporates corresponding human and/or mouse L-R pairs from CellTalkDB (Sup Table 1). Collectively, these improvements establish FlyPhoneDB2 as a more comprehensive resource for studying CCC in *Drosophila* and make it easy to identify the L-R pairs that are conserved in humans to provide insight about their potential mechanisms relevant to human health (Table 1).

**Table 1:** Summary of the major differences of FlyPhoneDB1 and FlyPhoneDB2.

**Information**	**FlyPhoneDB1**	**FlyPhoneDB2**
ligand count	96	393
receptor count	85	479
ligand-receptor pair count	196	1804
computational time sec. (7 K cells, 6 clusters, 1 core)	1140	35
visualization	dot plot, circle plot and heatmap	many more added including chord diagrams, scatter plot
pathway annotation	ligand, receptor	ligand, receptor, reporter
compare two samples?	no	yes
ligand annotation?	no	yes
ligand signal peptide prediction?	no	yes
receptor annotation?	no	yes
receptor TM prediction?	no	yes
mammalian LR pairs info?	no	yes

### 3.2 Improvement of the performance and output illustration

**Fig. 2:**
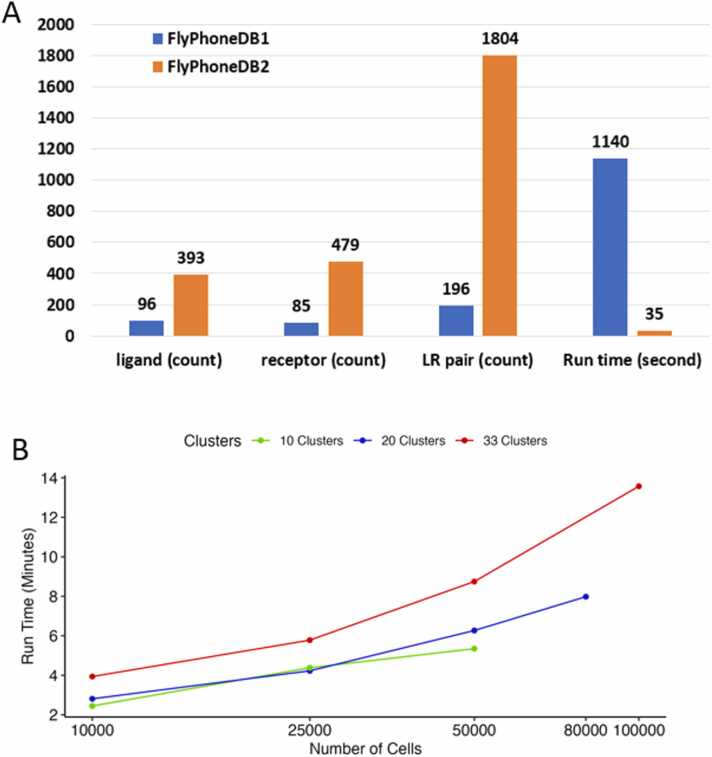
Comparison of FlyPhoneDB1 and FlyPhoneDB2. A) Summary of the improvement in database coverage and run time. The test dataset contains 7 K cells with 6 cell types. B) Run time comparison with different configuration. The datasets tested contain 50 K cells with 10 clusters, 80 K cells with 20 clusters and 100 K with 33 clusters, respectively. The tests were run on the datasets with cell numbers down sampled as well.

FlyPhoneDB evaluates each ligand-receptor interaction by assessing ligand expression in the source cell and receptor expression in the target cell, calculating an interaction score for each source–target cell pair for each L-R pair. The specificity and significance of each interaction score are then evaluated using a permutation test, where cell labels are shuffled to identify CCC events with significant p-values. With the expansion of the database to include hundreds of additional L-R pairs, it became necessary to improve the core pipeline to reduce computational time. We optimized the permutation step, the most time-consuming part of the pipeline, which resulted in a substantial reduction in computational time. For example, FlyPhoneDB2 demonstrates more than a 30-fold improvement in performance on a test dataset, completing analyses in seconds compared to minutes with the original FlyPhoneDB pipeline (Fig. 2, Sup Fig. 2A), without compromising results. Notably, 99 % of CCC events identified by FlyPhoneDB2 at a p-value cutoff of 0.05 are consistent with those identified by the original FlyPhoneDB, although consistency may vary slightly between different cell types due to the stochastic nature of permutation-based p-value calculation (Sup Fig. 2B). In addition, with the improved performance, parallel processing is no longer required with the new pipeline (Sup Fig. 2A).

**Fig. 3:**
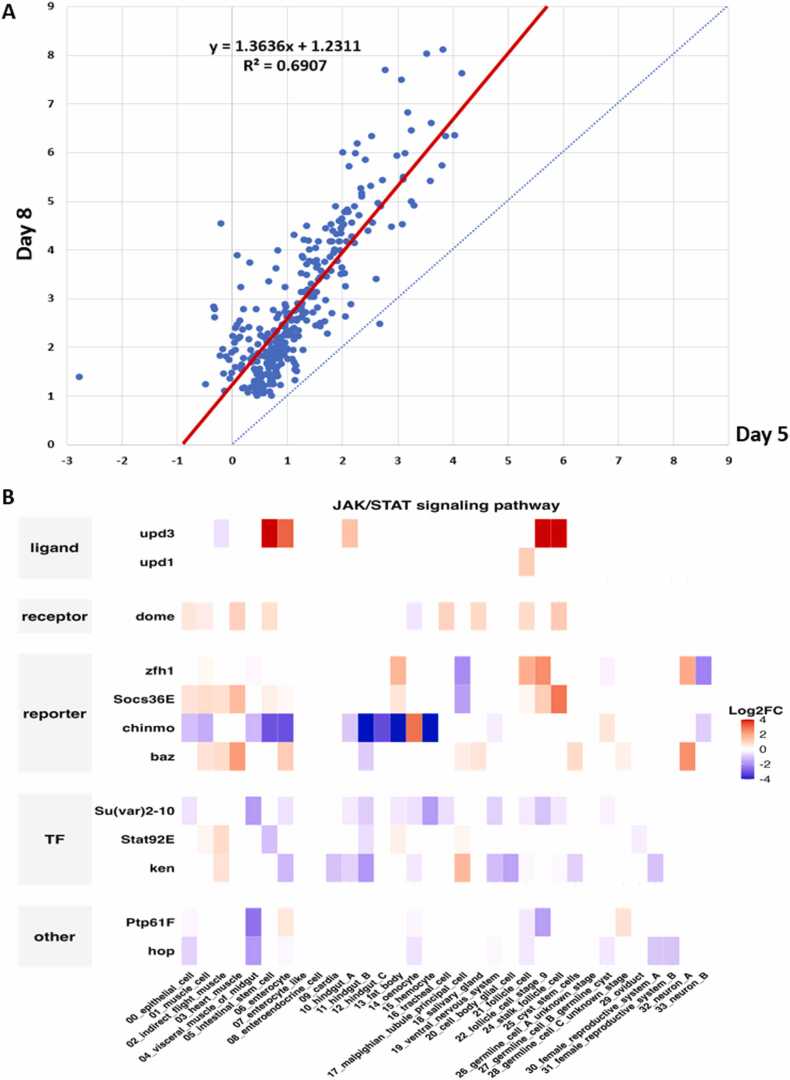
Analysis of scRNAseq data of adult full body from Yki fly using FlyPhoneDB2. A) Comparison and visualization of the differential scores of CCC activities between Yki tumor and corresponding wild type flies of day 5 samples with day 8 samples using scatter plot. The differential scores from the two time points correlate well (R^2^= 0.6907; Pearson correlation = 0.83) while much higher magnitude changes are observed in day 8 samples. B) Example of heatmap of expression changes in the core components from JAK-STAT signaling pathway of day 8 Yki tumor sample comparing to day 8 wild type control.

FlyPhoneDB2 offers a suite of both enhanced and entirely new visualizations that represent a significant advancement over the original FlyPhoneDB (Fig. 3, Sup Fig. 3–5). In addition to the original circle plots depicting the direction and strength of CCC, FlyPhoneDB2 introduces scatter plots summarizing the total incoming and outgoing CCC for each cell type. These allow users to rapidly identify which cell types are predominant signal senders or receivers (Sup Fig. 3–5). A major enhancement in FlyPhoneDB2 is the adoption of chord diagrams, which clearly illustrate the strength of outgoing CCC from each source cell type to its target cells. This visualization enables users to easily evaluate the influence of individual source cell types on specific recipient populations at a network level (Sup Fig. 3–5).

While the original FlyPhoneDB provided heatmaps to indicate the expression of core components involved in each signaling pathway, facilitating pathway activity assessment in receiving cells, FlyPhoneDB2 refines and expands this feature. It now offers more detailed summary illustrations that independently display the expression levels of ligands, receptors, and reporter genes. Such granularity supports a more nuanced evaluation of signaling pathway activity, since the mere co-expression of ligand and receptor does not always guarantee pathway activation under all conditions [25].

### 3.3 Expanding the function of FlyPhoneDB to consider downstream reporter genes of signaling pathways

Cell signaling, also known as signal transduction, serves a crucial function in biological systems by transmitting extracellular signals to modulate intracellular gene expression. This process generally begins when a ligand binds to a membrane-bound receptor, setting off a series of intercellular signaling events mediated by various kinases. These events ultimately influence the regulation of downstream gene expression by transcription factors. A majority of the tools developed for CCC analysis only considers the L-R expression while there are situations when both the ligand and receptor are present but the signaling pathway remains inactive which can occur due to various regulatory mechanisms, such as receptor desensitization, inhibitory proteins, or post-translational modifications [25]. Therefore, taking into consideration of the expression levels of downstream reporter genes besides doing the L-R gene analysis can help better understand the activity of signaling pathways. However, the annotation of such genes for the major signaling pathway are quite limited and with the aid from experts in the field, we assembled reporter gene list curated manually from literature, which covers 1–25 genes for various signaling pathways (Sup table 2). In addition to providing heatmaps of core component expression for each signaling pathway, as previously implemented, the new release also features heatmaps that display the average expression values of reporter genes per cell type, grouped by pathway. Since multiple reporter and receptor genes may be annotated for many pathways, we highlight the reporter and receptor genes with the highest expression levels. Their expression is summarized and visualized using a dot plot, where the color represents the highest expression level of reporter genes and the dot size indicates the highest expression level of receptor genes. This enables users to seamlessly navigate between ligand–receptor scores (based on ligand/receptor expression) and pathway activity (based on reporter gene expression in target cells), helping prioritize the most biologically relevant CCC events between different cell types.

### 3.4 Expanding the function of FlyPhoneDB to compare the CCC between samples

Investigating CCC in both healthy and disease states is increasingly important for elucidating mechanisms underlying disease development. FlyPhoneDB2 addresses this need by introducing enhanced functionality and exploratory visualizations specifically designed to facilitate such comparative analyses. Users can now specify samples within their dataset, enabling the pipeline to generate sample-specific expression matrices. The pipeline then analyzes CCC events for each sample, inferring cell type–specific signaling events within each context. Once individual analyses are complete, CCC event scores are compared across samples to identify events that differ between conditions. These differential CCC events are then selected and visualized for further exploration. Additionally, users have the option to upload a list of differentially expressed genes (DEGs), allowing for further optimization and filtering of communication events based on gene expression changes. FlyPhoneDB2 also provides dedicated visualizations to facilitate direct comparison between samples, including circle plots for differential CCC events (Sup Figure 5D) and heatmaps for differential expression of pathway core components (Fig. 3B, Sup Figure 4C,4D,5 C). This new function helps elucidate differences in CCC between different genotypes or conditions at the tissue, signaling pathway, and L-R pair level. The choice of candidate differential L-R pairs to visualize and use for downstream analysis is highly dependent on the biological question. Investigating potential perturbations in known signaling events between tissues may require strict filtering while exploratory analysis may benefit from more relaxed thresholds to capture a broader range of potential interactions.

### 3.5 Use case: tumor dependent communication events from an adult full body scRNAseq dataset

Cancer cachexia, characterized by progressive muscle and fat loss, is a major contributor to cancer-related mortality and severely impacts patients’ quality of life and treatment outcomes. Despite its prevalence in cancer patients, effective therapies remain elusive due to the syndrome’s complex, multi-organ nature. Therefore, understanding the underlying pathogenic mechanisms requires a detailed characterization of tumor-host organ communications.

A *Drosophila* model of cancer cachexia has been established by expressing an activated form of the Yorkie (Yki)/Yap oncogene in adult fly intestinal stem cells (ISC) (*esg>yki*^*act*^; hereafter referred to as Yki flies). At day 5 after tumor induction, tumors encompass most of the gut but the peripheral organ wasting is just beginning to emerge. By day 8, these symptoms become severe, evidenced by pronounced bloating (fluid accumulation), an indicator of cachexia in Yki flies [26], [27]. Therefore, tumor-host communications that persist from day 5 to day 8 are likely contributors to these phenotypes. Previous transcriptome analyses of Yki tumor-bearing guts have identified a number of cachexia-associated ligands, including Pvr1, ImpL2, and upd3 [26], [27], [28]. However, a comprehensive, unbiased analysis is still needed to identify additional potential cachexia ligands and to determine their systemic impact across the full body, including their target tissues. The transcriptomic data from adult full body (no head) of Yki flies at single nucleus resolution has been made available recently [23], including 122,898 cells from wild type and Yki flies at 5 days (25,146 control cells and 42,375 tumor cells) and 8 days (19,050 control cells and 36,327 tumor cells) after tumor induction.

Here, we present a case study demonstrating the use of FlyPhoneDB2 to identify tumor-host interactions underlying cancer cachexia using full-body scRNA-seq datasets. We retrieved raw data from GEO (accession GSE229526), and uploaded the expression matrices and metadata for each time point (wild-type and Yki tumor samples) into FlyPhoneDB2. Focusing on 910 medium/high-confidence L-R pairs from the updated knowledgebase, we analyzed CCC events across all four samples. The results were visualized using tables of L-R pair scores, along with signaling pathway activity illustrated by heatmaps, circle plots, and chord diagrams for each sample (Sup Fig. 3). FlyPhoneDB2 was also configured to directly compare wild-type and Yki tumor samples at each time point, enabling the identification of L-R pairs specifically associated with tumor progression. We selected CCC events that were significant in Yki samples but not in wild-type at day 5 and/or day 8 (p < 0.05), and further filtered for those with an interaction score difference > 2 (or >1 in on/off cases). To prioritize biologically relevant events, we focused on CCC events involving ligands that were differentially expressed in each time point, particularly those originating from tumor cell types (ISC or EC; Sup Table 3). This approach identified 110 up-regulated CCC events corresponding to 25 L-R pairs in day 5 tumor samples, and 615 up-regulated events for 102 L-R pairs at day 8. Among these, 35 ligands were found at day 8, of which 14 were also detected at day 5 (Sup Table 3). We observed a strong correlation in differential CCC events between time points, with greater magnitude changes at day 8 (Fig. 3A), consistent with the more severe phenotype of peripheral organ wasting at this stage. Notably, among the 35 ligands identified in the 615 CCC events, we observed increased upd3–dome signaling (Fig. 3B, Sup Figure 4 and 5) and Pvr1–Pvr signaling (Sup Figure 4 and 5), in agreement with previous studies [27], [28]. In addition, the analysis systematically identified the potential target tissues affected by these cachexia ligands through assessing the tissue-specific expression of receptors (Fig. 3B, Sup figure 4 and 5). For instance, FlyPhoneDB2 analysis point out that Pvf1 primarily targets Malpighian Tubules, which was demonstrated by a recent study charactering a pathogenic mechanism of paraneoplastic nephrotic syndrome in the Yki model [29].

In addition to previously characterized ligands, we identified 32 new ligands, including several ligands that were reported in other fly cancer cachexia models. For example, we identified matrix metalloproteinases (MMPs) and *branchless* (*bnl*) upregulation in Yki tumors. MMPs were reported to modulate TGF-β signaling in the fat body and disrupt basement membrane (BM)/extracellular matrix (ECM) protein localization in both the fat body and muscle, leading to muscle wasting [30]. *bnl* was identified as an inducer of muscle wasting in a high-sugar diet (HSD)-enhanced tumor model [31]. Importantly, our analysis revealed previously unstudied secreted factors, such as *dally-like* (*dlp*) and *slit* (*sli*). *sli* is a secreted glycoprotein and serves as the ligand for the Robo receptor family and co-receptors. Its human ortholog, SLIT2, has not been specifically implicated in cancer cachexia. However, studies have shown that a secreted fragment of SLIT2 regulates adipose tissue thermogenesis and metabolic function [32], suggesting that tumor-secreted SLIT2 may impair adipose tissue function, disrupting energy balance and contributing to cachexia. *dlp* regulates the signaling strength and range of *Hedgehog* (*Hh*) and *Wingless* (*Wg*). While aberrant Hedgehog signaling is known to support tumorigenesis [33], our data suggests that its role in cancer cachexia needs to be further explored.

Altogether, FlyPhoneDB2 represents a significant advancement in the identification of cachexia-associated secreted factors and the mapping of their target tissues at single-cell resolution. This approach deepens our understanding of the systemic impact of tumor-derived signals and establishes a foundation for mechanistic studies of tumor-induced organ dysfunction. By uncovering novel ligands and their receptor interactions, FlyPhoneDB2 also enables the generation of new hypotheses regarding inter-organ crosstalk in cancer cachexia. Overall, this example highlights the power of FlyPhoneDB2 to reveal dysregulated L-R signaling pathways and to provide candidate pathways for further validation.

## 4 Concluding remarks

CCC and signaling pathways are fundamental to the proper functioning of biological systems, enabling cells to coordinate their activities, respond to environmental changes, and maintain homeostasis. Bioinformatics tools for analyzing CCC are becoming increasingly powerful and versatile, driven by technological advances and the growing complexity of biological data. FlyPhoneDB2 builds upon its core algorithm to generate valuable biological insights from scRNA-seq data, elucidating CCC between diverse cell types. The updated algorithm offers increased computational speed and facilitates the discovery of dysregulated signaling under different conditions (Table 1). To date, 1804 L-R pairs have been annotated in the FlyPhoneDB2 database, with AI-predicted and community-submitted L-R pairs continually validated and added. The use case analyzing signaling events in full-body scRNA-seq data from the Yki tumor model demonstrates the power of FlyPhoneDB2 to uncover dysregulated L-R signaling pathways and provide candidate pathways for further validation.

We will continually update and expand the FlyPhoneDB2 knowledgebase as new experimental evidence emerges and additional L-R pairs are predicted using AlphaFold-Multimer. Looking ahead, the future of tool development in this field lies in the integration of multi-omics data, the application of AI, and the provision of spatial and dynamic insights. Together, these advances will further enhance our understanding of cellular interactions in both health and disease.

## CRediT authorship contribution statement

**Myeonghoon Han:** Writing – review & editing, Software, Data curation. **Eric Zhou:** Visualization, Software. **Tzu-Chiao Lu:** Data curation. **Hongjie Li:** Writing – review & editing, Data curation. **Norbert Perrimon:** Writing – review & editing, Supervision, Resources, Funding acquisition, Conceptualization. **Austin Veal:** Software. **Yanhui Hu:** Writing – review & editing, Writing – original draft, Visualization, Validation, Supervision, Project administration, Methodology, Formal analysis, Data curation, Conceptualization. **Mujeeb Qadiri:** Writing – review & editing, Writing – original draft, Visualization, Software, Methodology, Formal analysis. **Ying Liu:** Writing – review & editing, Visualization, Validation, Methodology, Formal analysis, Data curation. **Ah-Ram Kim:** Writing – review & editing, Software, Data curation.

## Conflict of Interest

The authors declare no conflicts of interest.

## Data Availability

The resource is available to pubic and the online version is at https://www.flyrnai.org/tools/fly_phone_v2, and FlyPhoneDB2 analysis results of day 5 and day 8 Yki datasets are available at https://www.flyrnai.org/tools/fly_phone_v2/web/yorkie_tumor_analysis. The code of standalone version is available at GitHub (https://github.com/FullStackGoogler/FlyPhoneDB2). AlphaFold-Multimer prediction results used in this study are available at the FlyPredictome (https://www.flyrnai.org/tools/fly_predictome). This article is subject to HHMI’s Open Access to Publications policy. HHMI lab heads have previously granted a nonexclusive CC BY 4.0 license to the public and a sublicensable license to HHMI in their research articles. Pursuant to those licenses, the author-accepted manuscript of this article can be made freely available under a CC BY 4.0 license immediately upon publication.

## References

[1] Efremova M., Vento-Tormo M., Teichmann S.A., Vento-Tormo R. (2020). CellPhoneDB: inferring cell-cell communication from combined expression of multi-subunit ligand-receptor complexes. Nat Protoc.

[2] Jin S., Guerrero-Juarez C.F., Zhang L., Chang I., Ramos R., Kuan C.H. (2021). Inference and analysis of cell-cell communication using CellChat. Nat Commun.

[3] Svensson V., Teichmann S.A., Stegle O. (2018). SpatialDE: identification of spatially variable genes. Nat Methods.

[4] Dries R., Zhu Q., Dong R., Eng C.L., Li H., Liu K. (2021). Giotto: a toolbox for integrative analysis and visualization of spatial expression data. Genome Biol.

[5] Liu Y., Li J.S.S., Rodiger J., Comjean A., Attrill H., Antonazzo G. (2022). FlyPhoneDB: an integrated web-based resource for cell-cell communication prediction in Drosophila. Genetics.

[6] Raz A.A., Vida G.S., Stern S.R., Mahadevaraju S., Fingerhut J.M., Viveiros J.M. (2023). Emergent dynamics of adult stem cell lineages from single nucleus and single cell RNA-Seq of Drosophila testes. Elife.

[7] Xu J., Liu Y., Li H., Tarashansky A.J., Kalicki C.H., Hung R.J. (2022). Transcriptional and functional motifs defining renal function revealed by single-nucleus RNA sequencing. Proc Natl Acad Sci USA.

[8] Park Y.J., Lu T.C., Jackson T., Goodman L.D., Ran L., Chen J. (2024). Whole organism snRNA-seq reveals systemic peripheral changes in Alzheimer's Disease fly models. bioRxiv.

[9] Bukhari H., Nithianandam V., Battaglia R.A., Cicalo A., Sarkar S., Comjean A. (2024). Transcriptional programs mediating neuronal toxicity and altered glial-neuronal signaling in a Drosophila knock-in tauopathy model. Genome Res.

[10] Shao X., Liao J., Li C., Lu X., Cheng J., Fan X. (2021). CellTalkDB: a manually curated database of ligand-receptor interactions in humans and mice. Brief Bioinform.

[11] Hu Y., Flockhart I., Vinayagam A., Bergwitz C., Berger B., Perrimon N. (2011). An integrative approach to ortholog prediction for disease-focused and other functional studies. BMC Bioinforma.

[12] Hallgren J., Tsirigos K.D., Pedersen M.D., Almagro Armenteros J.J., Marcatili P., Nielsen H., et al. DeepTMHMM predicts alpha and beta transmembrane proteins using deep neural networks. bioRxiv. 2022:2022.04.08.487609. doi: 10.1101/2022.04.08.487609.

[13] Krogh A., Larsson B., von Heijne G., Sonnhammer E.L. (2001). Predicting transmembrane protein topology with a hidden Markov model: application to complete genomes. J Mol Biol.

[14] Braun P., Tasan M., Dreze M., Barrios-Rodiles M., Lemmens I., Yu H. (2009). An experimentally derived confidence score for binary protein-protein interactions. Nat Methods.

[15] Tang H.W., Spirohn K., Hu Y., Hao T., Kovacs I.A., Gao Y. (2023). Next-generation large-scale binary protein interaction network for Drosophila melanogaster. Nat Commun.

[16] Yu H., Braun P., Yildirim M.A., Lemmens I., Venkatesan K., Sahalie J. (2008). High-quality binary protein interaction map of the yeast interactome network. Science.

[17] Kim A.-R., Hu Y., Comjean A., Rodiger J., Mohr S.E., Perrimon N. Enhanced Protein-Protein Interaction Discovery via AlphaFold-Multimer. bioRxiv. 2024:2024.02.19.580970. doi: 10.1101/2024.02.19.580970.

[18] Subramanian A., Tamayo P., Mootha V.K., Mukherjee S., Ebert B.L., Gillette M.A. (2005). Gene set enrichment analysis: a knowledge-based approach for interpreting genome-wide expression profiles. Proc Natl Acad Sci USA.

[19] Honig B., Shapiro L. (2020). Adhesion protein structure, molecular affinities, and principles of cell-cell recognition. Cell.

[20] Togashi H., Sakisaka T., Takai Y. (2009). Cell adhesion molecules in the central nervous system. Cell Adh Migr.

[21] Hu Y., Vinayagam A., Nand A., Comjean A., Chung V., Hao T. (2018). Molecular Interaction Search Tool (MIST): an integrated resource for mining gene and protein interaction data. Nucleic Acids Res.

[22] Banhos Danneskiold-Samsoe N., Kavi D., Jude K.M., Nissen S.B., Wat L.W., Coassolo L. (2024). AlphaFold2 enables accurate deorphanization of ligands to single-pass receptors. Cell Syst.

[23] Liu Y., Dantas E., Ferrer M., Liu Y., Comjean A., Davidson E.E. (2023). Tumor cytokine-induced hepatic gluconeogenesis contributes to cancer cachexia: insights from full body single nuclei sequencing. bioRxiv.

[24] Hu Y., Ewen-Campen B., Comjean A., Rodiger J., Mohr S.E., Perrimon N. (2022). Paralog Explorer: A resource for mining information about paralogs in common research organisms. Comput Struct Biotechnol J.

[25] Pierce K.L., Premont R.T., Lefkowitz R.J. (2002). Seven-transmembrane receptors. Nat Rev Mol Cell Biol.

[26] Kwon Y., Song W., Droujinine I.A., Hu Y., Asara J.M., Perrimon N. (2015). Systemic organ wasting induced by localized expression of the secreted insulin/IGF antagonist ImpL2. Dev Cell.

[27] Song W., Kir S., Hong S., Hu Y., Wang X., Binari R. (2019). Tumor-derived ligands trigger tumor growth and host wasting via differential MEK activation. Dev Cell.

[28] Ding G., Xiang X., Hu Y., Xiao G., Chen Y., Binari R. (2021). Coordination of tumor growth and host wasting by tumor-derived Upd3. Cell Rep.

[29] Xu J., Liu Y., Yang F., Cao Y., Chen W., Li J.S.S. (2024). Mechanistic characterization of a Drosophila model of paraneoplastic nephrotic syndrome. Nat Commun.

[30] Lodge W., Zavortink M., Golenkina S., Froldi F., Dark C., Cheung S. (2021). Tumor-derived MMPs regulate cachexia in a Drosophila cancer model. Dev Cell.

[31] Newton H., Wang Y.F., Camplese L., Mokochinski J.B., Kramer H.B., Brown A.E.X. (2020). Systemic muscle wasting and coordinated tumour response drive tumourigenesis. Nat Commun.

[32] Svensson K.J., Long J.Z., Jedrychowski M.P., Cohen P., Lo J.C., Serag S. (2016). A secreted Slit2 fragment regulates adipose tissue thermogenesis and metabolic function. Cell Metab.

[33] Cochrane C.R., Szczepny A., Watkins D.N., Cain J.E. (2015). Hedgehog signaling in the maintenance of cancer stem cells. Cancers (Basel).

