# Supplementary material for: FlyPhoneDB2: A computational framework for analyzing cell-cell communication in Drosophila scRNA-seq data integrating AlphaFold-multimer predictions
Source: Comput Struct Biotechnol J. 2025 Jun 20;27:2814–22. doi: 10.1016/j.csbj.2025.06.032 (PMC12269837; doi:10.1016/j.csbj.2025.06.032)
Supplement: Supplementary file 1 — Supplementary material [file mmc1.pdf]

Sup Figure1: Overview of AlphaFold-Multimer Predictions for *Drosophila* Ligand–Receptor Pairs.
(A) Rank-ordered distributions of the five metrics for the curated ligand–receptor pairs from the original FlyPhoneDB. Blue points are individual complexes; the dashed red line marks the 10 % FPR threshold (value in panel), and the inset shows the percentage of pairs above that cut-off. Metrics, left-to-right, are: √(LIS × cLIS), LIS, cLIS, ipTM, Confidence, ipTM, Confidence
(B) Heatmap summarizing which metrics each pair passes (blue = ≥ cut-off; light gray = < cut-off), with the composite “Score” row below (green gradient = number of metrics passed, 0–5). Columns are sorted by descending Score. The side legend shows the pass/fail colors and the score gradient.
(C) Representative high-confidence complexes predicted by AlphaFold-Multimer for four signaling pathways. Ligand (purple cartoon/surface) and receptor (orange cartoon/surface) interacting domains are highlighted. From left to right: InR$^{674-1052}$ with Ilp2$^{26-137}$ (insulin signaling), Fz$^{41-171}$ with Wg$^{67-468}$ (Wnt signaling), Htl$^{235-428}$ with Bnl$^{80-573}$ (FGF signaling), Dome$^{17-523}$ with Upd2$^{109-355}$ (JAK/STAT signaling).

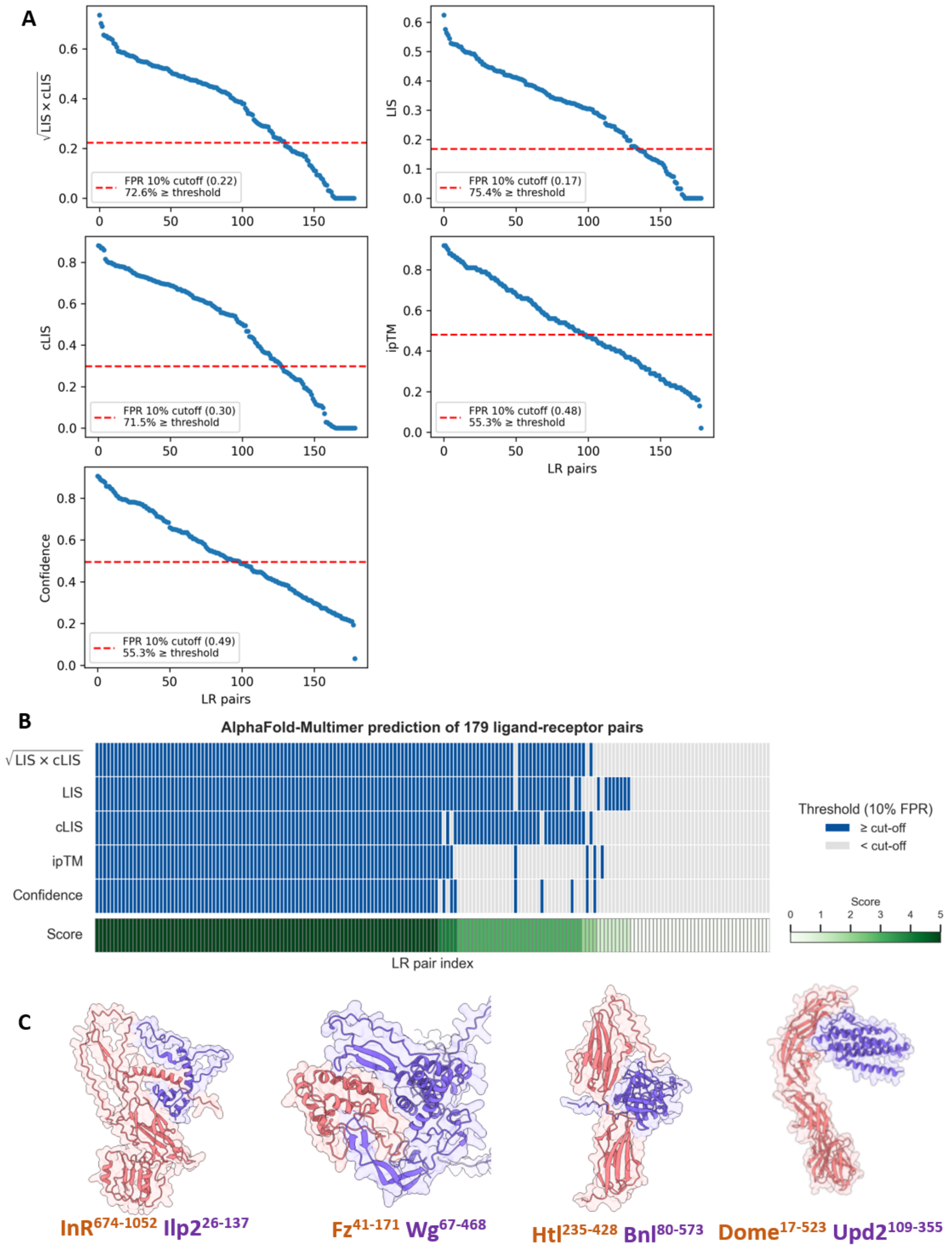

A
√LIS × cLIS
FPR 10% cutoff (0.22)
72.6% ≥ threshold
LIS
FPR 10% cutoff (0.17)
75.4% ≥ threshold
cLIS
FPR 10% cutoff (0.30)
71.5% ≥ threshold
ipTM
FPR 10% cutoff (0.48)
55.3% ≥ threshold
LR pairs
Confidence
FPR 10% cutoff (0.49)
55.3% ≥ threshold
LR pairs
B
AlphaFold-Multimer prediction of 179 ligand-receptor pairs
√LIS × cLIS
LIS
cLIS
ipTM
Confidence
Score
LR pair index
Threshold (10% FPR)
≥ cut-off
< cut-off
Score
C
InR674-1052 Ilp226-137
Fz41-171 Wg67-468
Htl235-428 Bnl80-573
Dome17-523 Upd2109-355

Sup Figure2: FlyPhoneDB2 demonstrates significant improvement in computational time without compromising the results on a test dataset.
(A) Run-time performance comparison of FlyPhoneDB2 vs FlyPhoneDB with various run-time parameters.
(B) A bar plot showing the percentage of CCC events in FlyPhoneDB which were also found in FlyPhoneDB2.

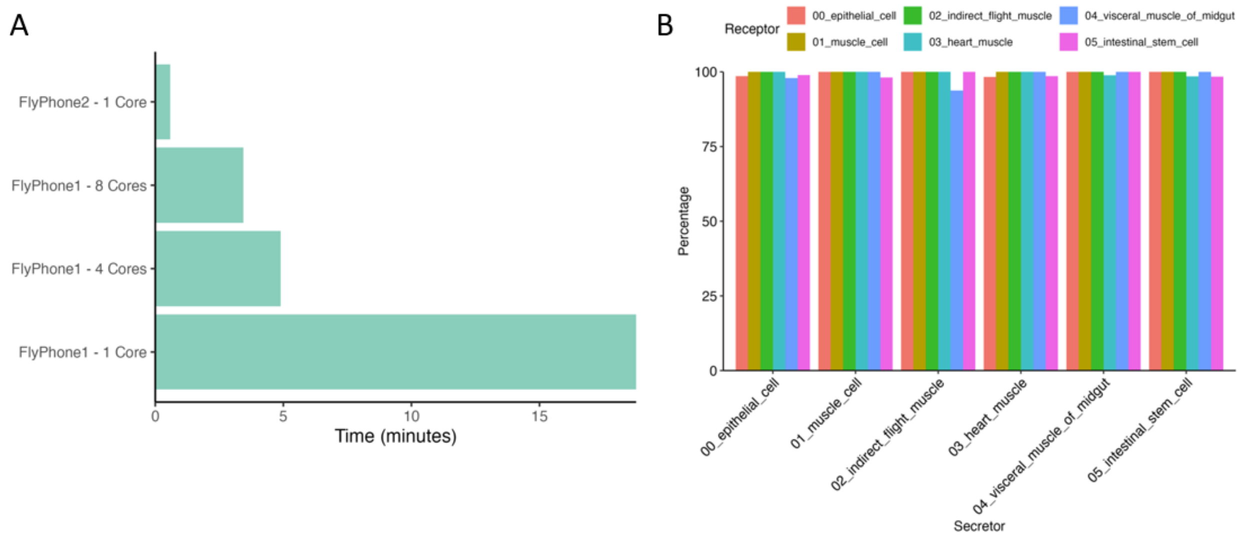

Sup Figure3: FlyPhoneDB2 is available as a web tool for users to upload data as well as a standalone program. FlyPhoneDB2 provides result tables with CCC scores and P-values as well as various plots for visualization.

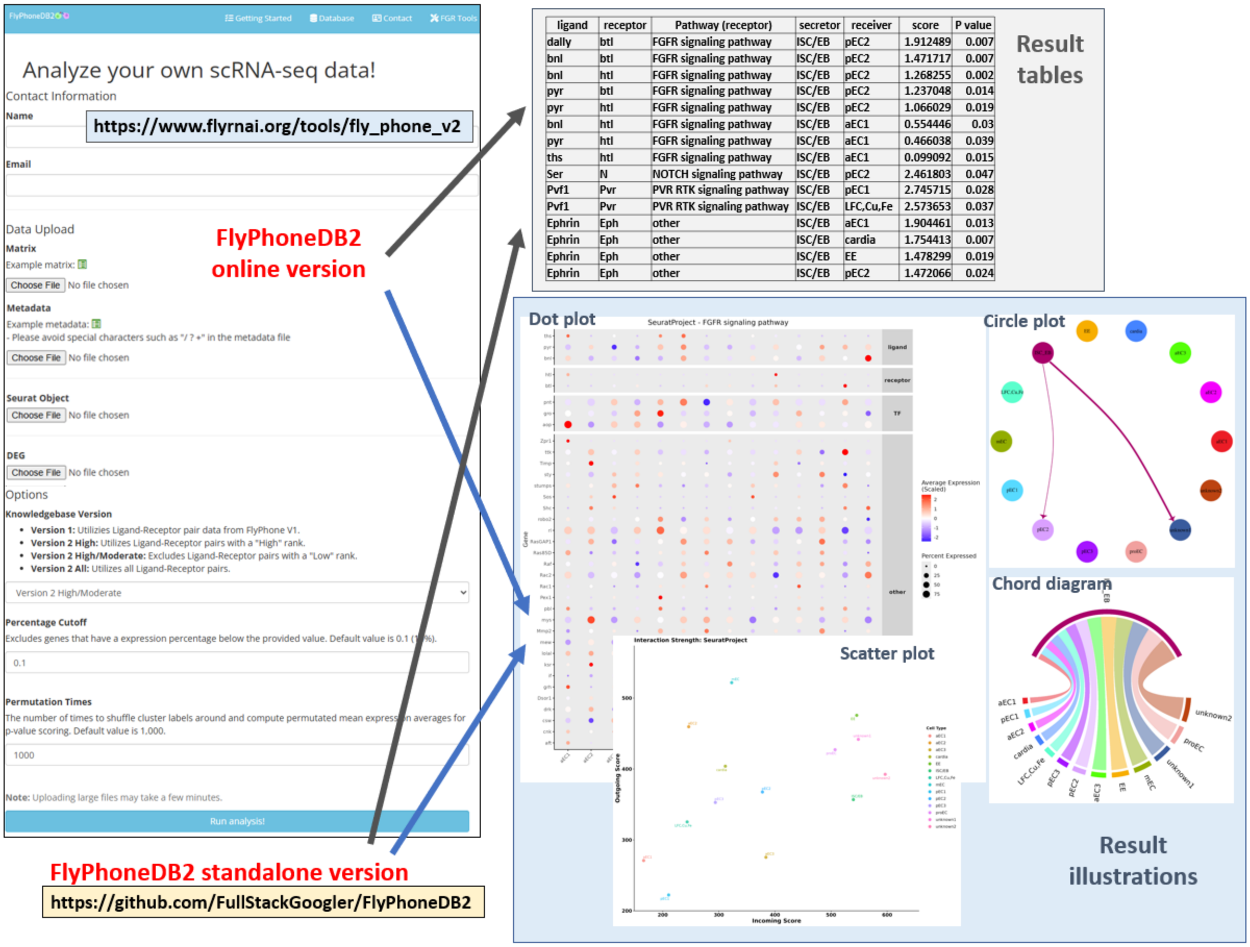

ligand	receptor	Pathway (receptor)	secretor	receiver	score	P value
dally	btl	FGFR signaling pathway	ISC/EB	pEC2	1.912489	0.007
bnl	btl	FGFR signaling pathway	ISC/EB	pEC2	1.471717	0.007
bnl	htl	FGFR signaling pathway	ISC/EB	pEC2	1.268255	0.002
pyr	btl	FGFR signaling pathway	ISC/EB	pEC2	1.237048	0.014
pyr	htl	FGFR signaling pathway	ISC/EB	pEC2	1.066029	0.019
bnl	htl	FGFR signaling pathway	ISC/EB	aEC1	0.554446	0.03
pyr	htl	FGFR signaling pathway	ISC/EB	aEC1	0.466038	0.039
ths	htl	FGFR signaling pathway	ISC/EB	aEC1	0.099092	0.015
Ser	N	NOTCH signaling pathway	ISC/EB	pEC2	2.461803	0.047
Pvf1	Pvr	PVR RTK signaling pathway	ISC/EB	pEC1	2.745715	0.028
Pvf1	Pvr	PVR RTK signaling pathway	ISC/EB	LFC,Cu,Fe	2.573653	0.037
Ephrin	Eph	other	ISC/EB	aEC1	1.904461	0.013
Ephrin	Eph	other	ISC/EB	cardia	1.754413	0.007
Ephrin	Eph	other	ISC/EB	EE	1.478299	0.019
Ephrin	Eph	other	ISC/EB	pEC2	1.472066	0.024

Sup Figure4: FlyPhoneDB2 analysis of day 5 Yki dataset with examples of data visualization.
(A) Scatter plot summarizing the total income and outgoing signals per cell type for both wild type and Yki data samples.
(B) Example of chord diagram for ISC cells regarding the strength of outgoing signals to various target cells.
(C) Example heatmap of expression changes in the core components from JAK-STAT signaling pathway of Yki tumor sample comparing to wild type control.
(D) Example heatmap of expression changes in the core components from Pvr RTK signaling pathway of Yki tumor sample comparing to wild type control.

A.

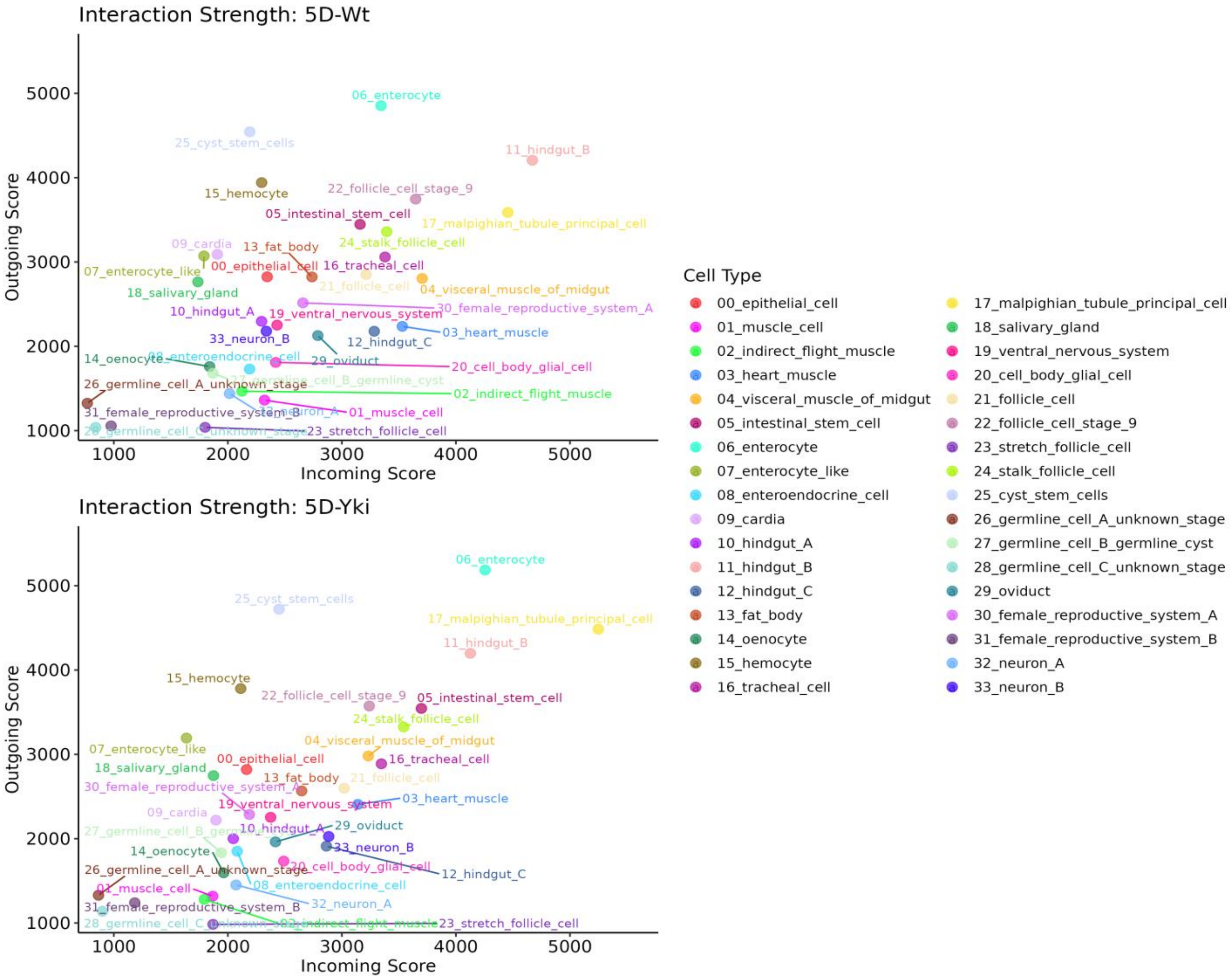

B.

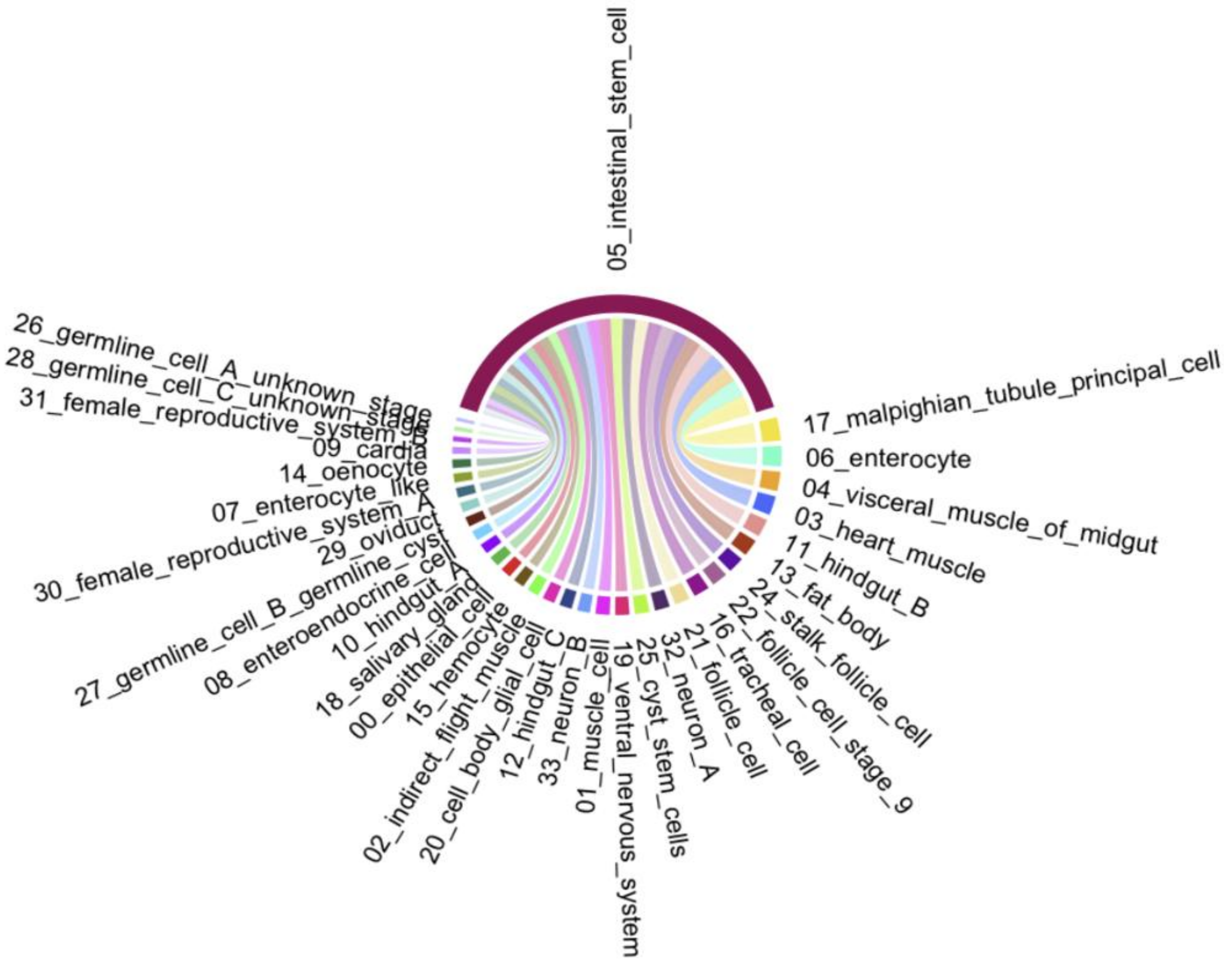

05_intestinal_stem_cell
17_malpighian_tubule_principal_cell
06_enterocyte
04_visceral_muscle_of_midgut
03_heart_muscle
11_hindgut_B
13_fat_body
24_stalk_follicle_cell
22_follicle_cell_stage_9
16_tracheal_cell
21_follicle_cell
32_neuron_A
25_cyst_stem_cells
19_ventral_nervous_system
01_muscle_cell
33_neuron_B
12_hindgut_C
20_cell_body_glial_cell
02_indirect_flight_muscle
15_hemocyte
00_epithelial_cell
18_salivary_gland
10_hindgut_A
08_enteroendocrine_cell
27_germline_cell_B_germline_cyst
29_oviduct
30_female_reproductive_system_A
07_enterocyte_like
14_oenocyte
09_cardia
31_female_reproductive_system_B
28_germline_cell_C_unknown_stage
26_germline_cell_A_unknown_stage

C.

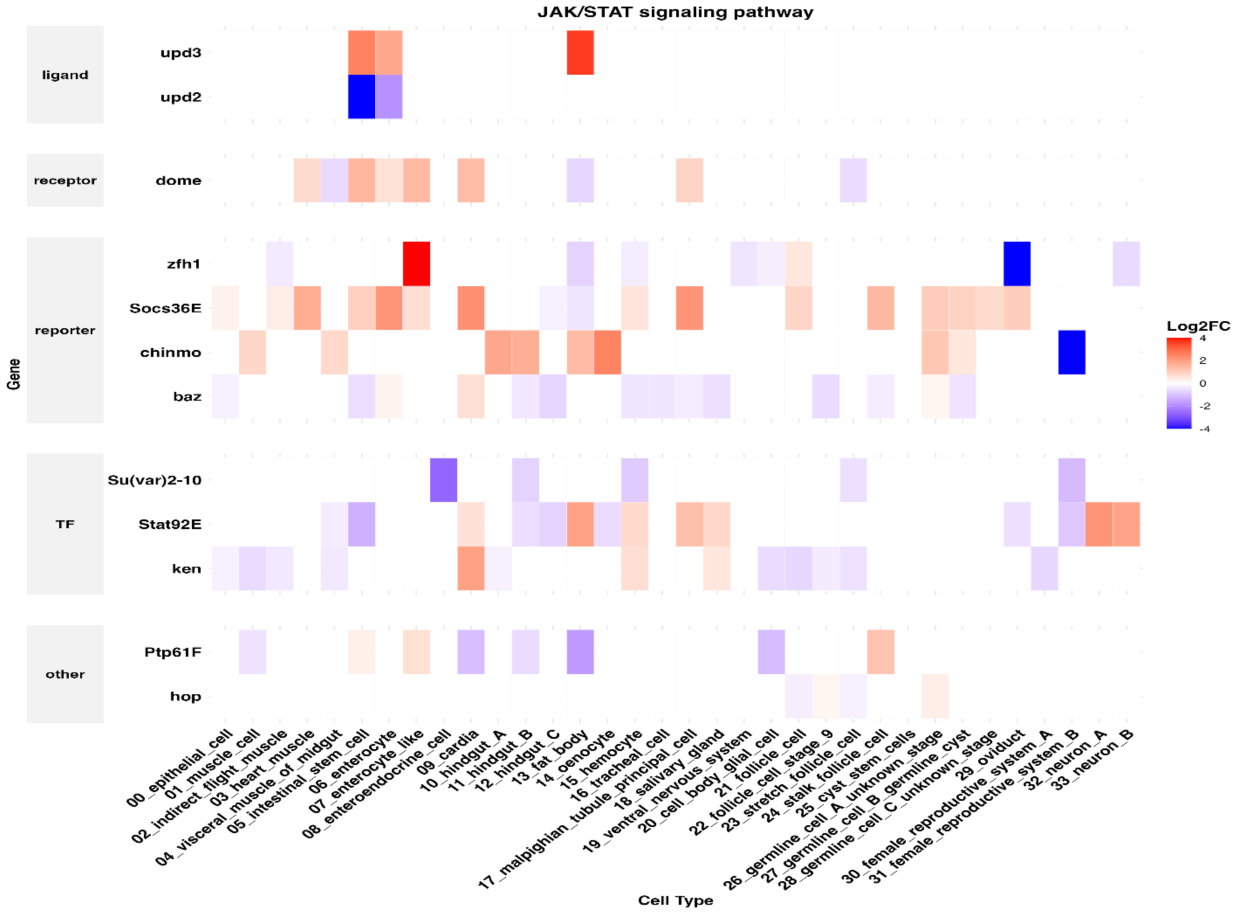
JAK/STAT signaling pathway
ligand
upd3
upd2
receptor
dome
reporter
zfh1
Socs36E
chinmo
baz
TF
Su(var)2-10
Stat92E
ken
other
Ptp61F
hop
Gene
Log2FC
4
2
0
-2
-4
00_epithelial_cell
01_muscle_cell
02_indirect_flight_muscle
03_heart_muscle
04_visceral_muscle_of_midgut
05_intestinal_stem_cell
06_enterocyte
07_enterocyte_like
08_enteroendocrine_cell
09_cardia
10_hindgut_A
11_hindgut_B
12_hindgut_C
13_fat_body
14_oenocyte
15_hemocyte
16_tracheal_cell
17_malpighian_tubule_principal_cell
18_salivary_gland
19_ventral_nervous_system
20_cell_body_glial_cell
21_follicle_cell
22_follicle_cell_stage_9
23_stretch_follicle_cell
24_stalk_follicle_cell
25_cyst_stem_cells
26_germline_cell_A_unknown_stage
27_germline_cell_B_germline_cyst
28_germline_cell_C_unknown_stage
29_oviduct
30_female_reproductive_system_A
31_female_reproductive_system_B
32_neuron_A
33_neuron_B
Cell Type

D.

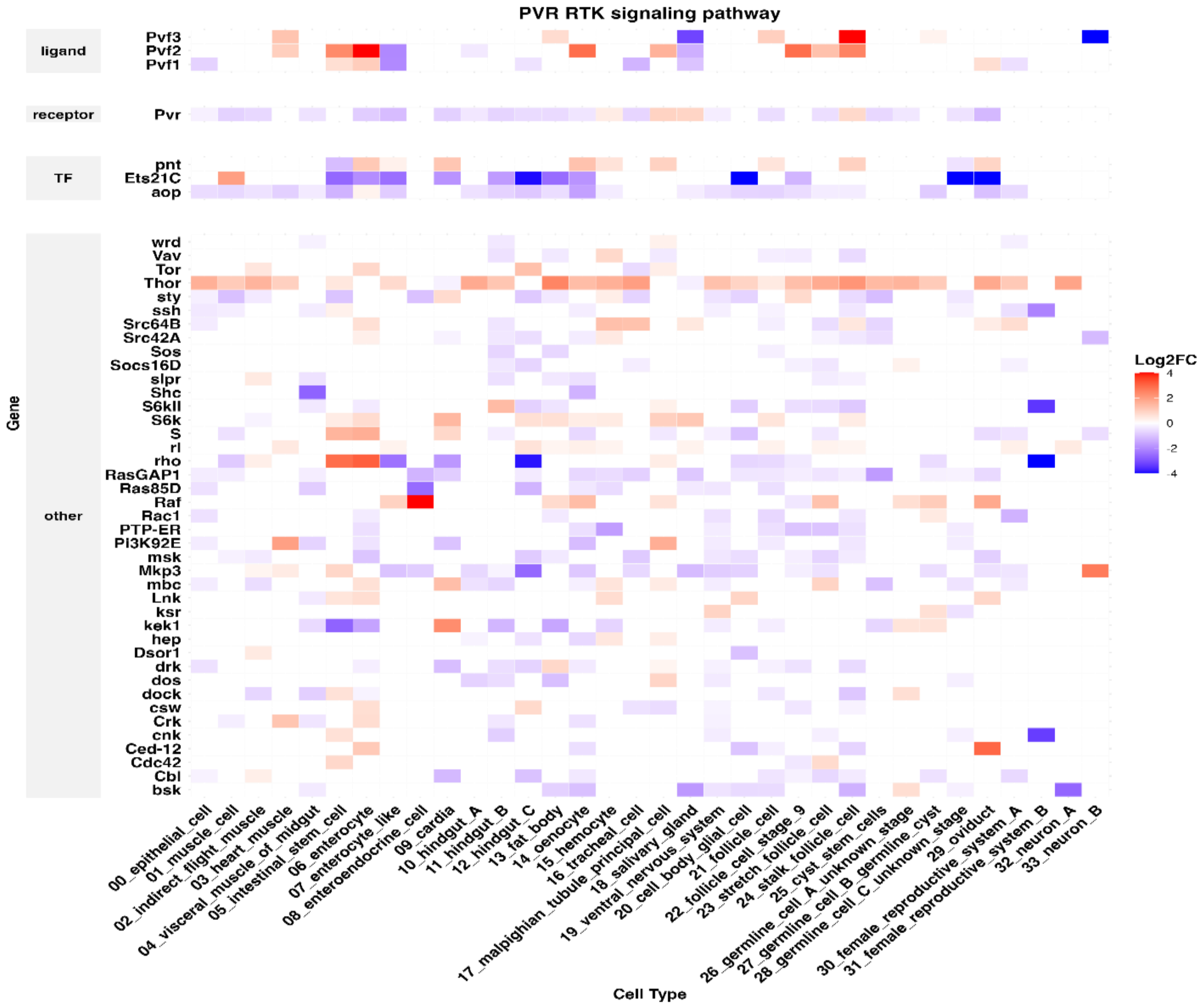

PVR RTK signaling pathway
ligand
Pvf3
Pvf2
Pvf1
receptor
Pvr
TF
pnt
Ets21C
aop
other
wrd
Vav
Tor
Thor
sty
ssh
Src64B
Src42A
Sos
Socs16D
slpr
Shc
S6kII
S6k
S
rl
rho
RasGAP1
Ras85D
Raf
Rac1
PTP-ER
PI3K92E
msk
Mkp3
mbc
Lnk
ksr
kek1
hep
Dsor1
drk
dos
dock
csw
Crk
cnk
Ced-12
Cdc42
Cbl
bsk
Gene
Log2FC
4
2
0
-2
-4
00_epithelial_cell
01_muscle_cell
02_indirect_flight_muscle
03_heart_muscle
04_visceral_muscle_of_midgut
05_intestinal_stem_cell
06_enterocyte
07_enterocyte_like
08_enteroendocrine_cell
09_cardia
10_hindgut_A
11_hindgut_B
12_hindgut_C
13_fat_body
14_oenocyte
15_hemocyte
16_tracheal_cell
17_malpighian_tubule_principal_cell
18_salivary_gland
19_ventral_nervous_system
20_cell_body_glial_cell
21_follicle_cell
22_follicle_cell_stage_9
23_stretch_follicle_cell
24_stalk_follicle_cell
25_cyst_stem_cells
26_germline_cell_A_unknown_stage
27_germline_cell_B_germline_cyst
28_germline_cell_C_unknown_stage
29_oviduct
30_female_reproductive_system_A
31_female_reproductive_system_B
32_neuron_A
33_neuron_B
Cell Type

Sup Figure5: FlyPhoneDB2 analysis of day 8 Yki dataset with examples of data visualization.
(A) Scatter plot summarizing the total income and outgoing signals per cell type for both wild type and Yki data samples.
(B) Example of chord diagram for ISC cells regarding the strength of outgoing signals to various target cells.
(C) Example heatmap of expression changes in the core components from Pvr RTK signaling pathway of Yki tumor sample comparing to wild type control.
(D) Example of circle plot of differential CCC in PVR RTK signaling pathway from intestinal stem cells (ISC).

A.

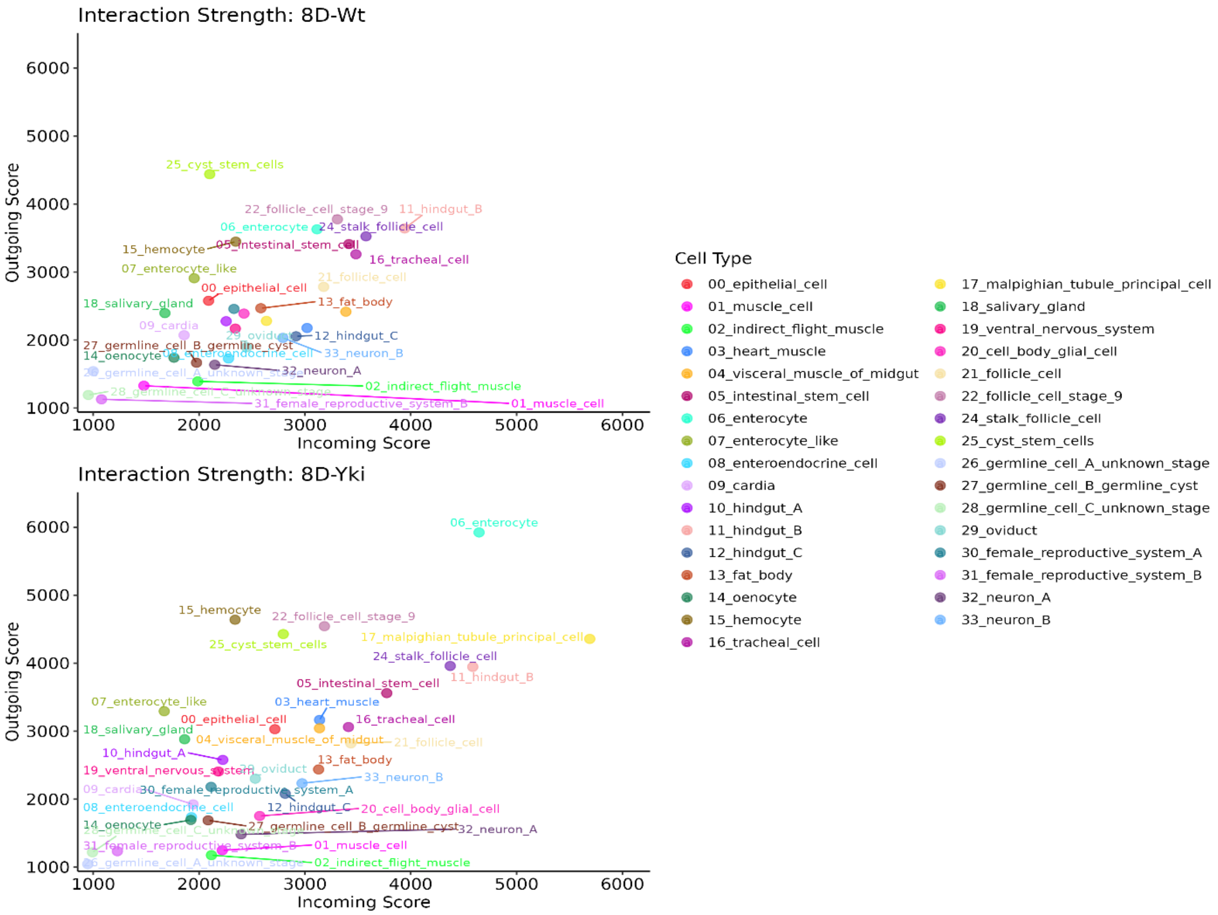

B.

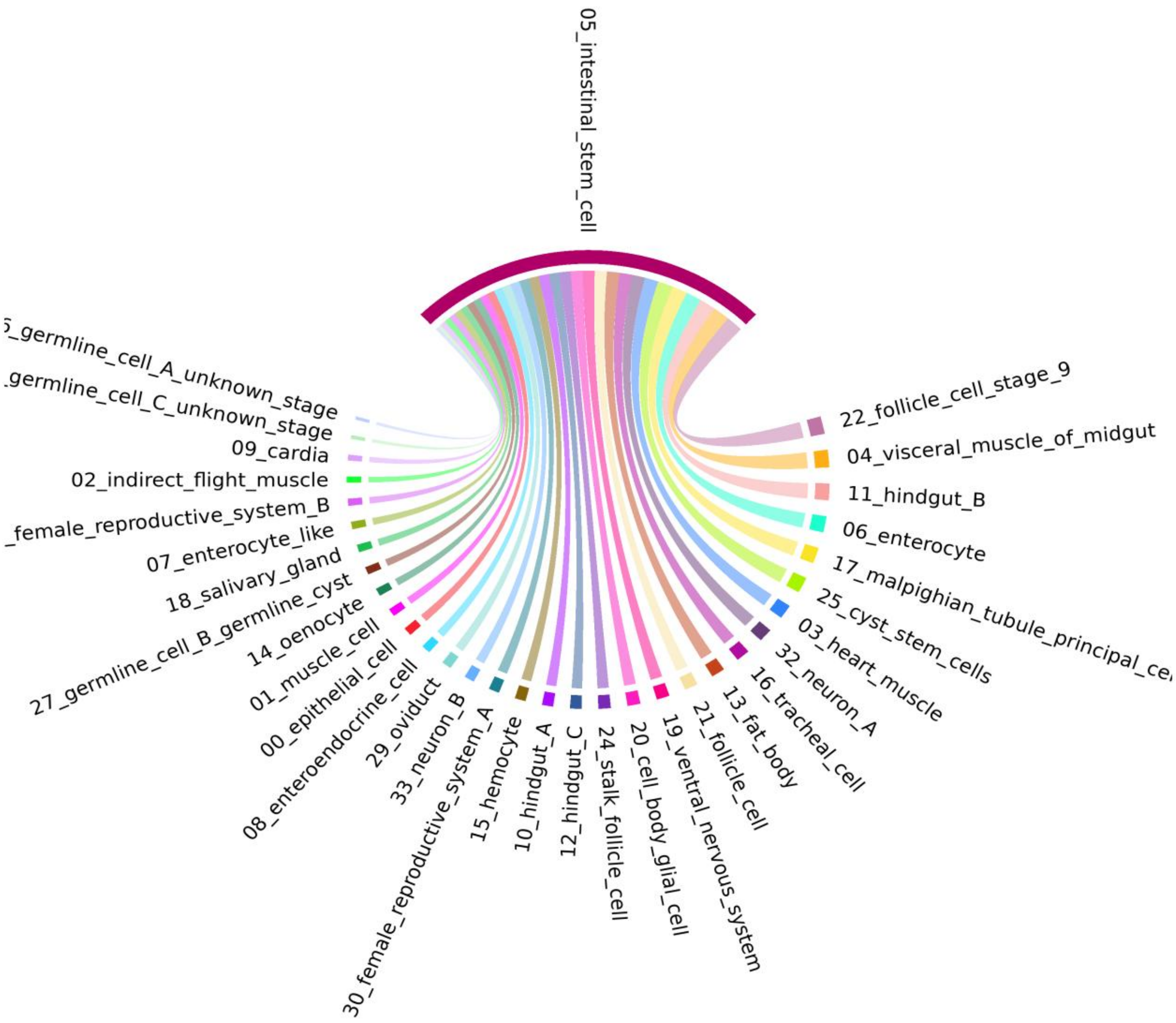
05_intestinal_stem_cell
_germline_cell_A_unknown_stage
germline_cell_C_unknown_stage
09_cardia
02_indirect_flight_muscle
_female_reproductive_system_B
07_enterocyte_like
18_salivary_gland
27_germline_cell_B_germline_cyst
14_oenocyte
01_muscle_cell
00_epithelial_cell
08_enteroendocrine_cell
29_oviduct
33_neuron_B
30_female_reproductive_system_A
15_hemocyte
10_hindgut_A
12_hindgut_C
24_stalk_follicle_cell
20_cell_body_glial_cell
19_ventral_nervous_system
21_follicle_cell
13_fat_body
16_tracheal_cell
32_neuron_A
03_heart_muscle
25_cyst_stem_cells
17_malpighian_tubule_principal_ce
06_enterocyte
11_hindgut_B
04_visceral_muscle_of_midgut
22_follicle_cell_stage_9

C.

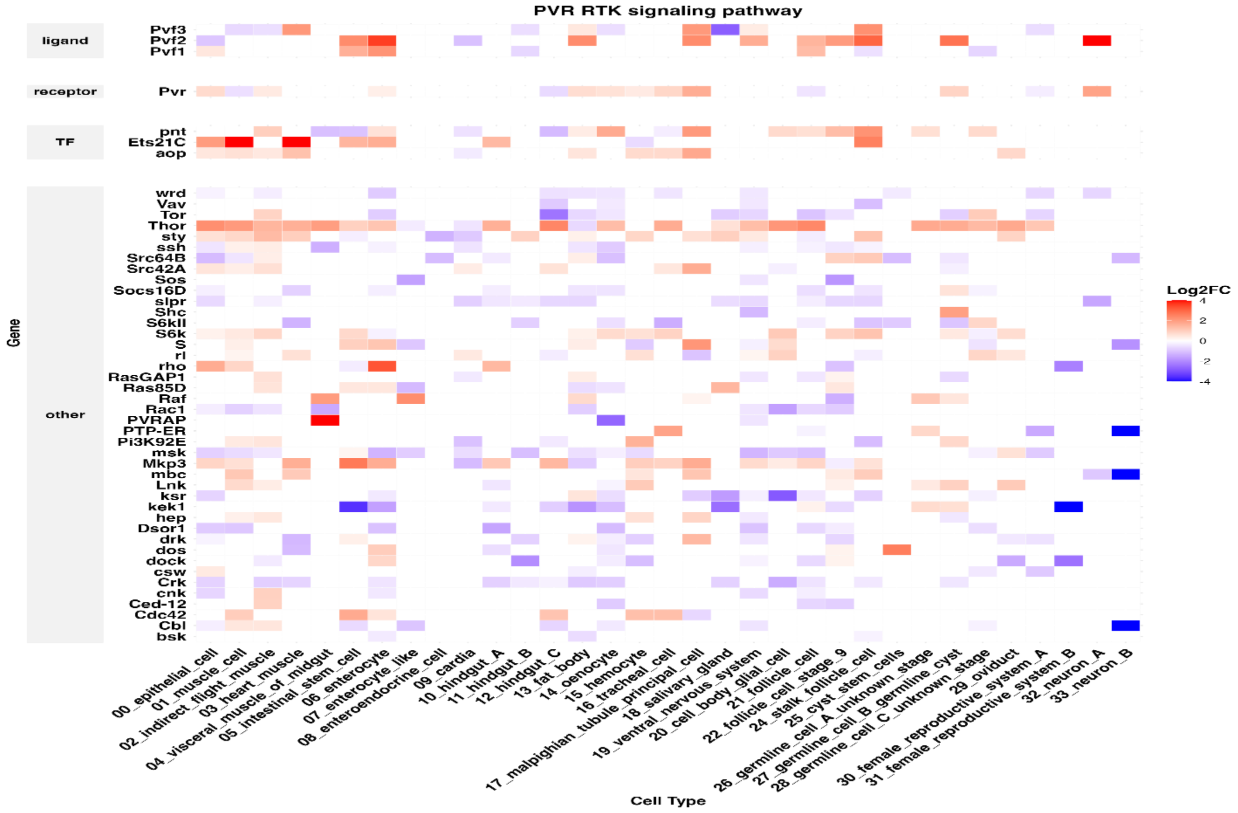
PVR RTK signaling pathway
ligand
receptor
TF
other
Gene
Cell Type
Log2FC
Pvf3
Pvf2
Pvf1
Pvr
pnt
Ets21C
aop
wrd
Vav
Tor
Thor
sty
ssh
Src64B
Src42A
Sos
Socs16D
slpr
Shc
S6kII
S6k
S
rl
rho
RasGAP1
Ras85D
Raf
Rac1
PVRAP
PTP-ER
Pi3K92E
msk
Mkp3
mbc
Lnk
ksr
kek1
hep
Dsor1
drk
dos
dock
csw
Crk
cnk
Ced-12
Cdc42
Cbl
bsk
00_epithelial_cell
01_muscle_cell
02_indirect_flight_muscle
03_heart_muscle
04_visceral_muscle_of_midgut
05_intestinal_stem_cell
06_enterocyte
07_enterocyte_like
08_enteroendocrine_cell
09_cardia
10_hindgut_A
11_hindgut_B
12_hindgut_C
13_fat_body
14_oenocyte
15_hemocyte
16_tracheal_cell
17_malpighian_tubule_principal_cell
18_salivary_gland
19_ventral_nervous_system
20_cell_body_glial_cell
21_follicle_cell
22_follicle_cell_stage_9
24_stalk_follicle_cell
25_cyst_stem_cells
26_germline_cell_A_unknown_stage
27_germline_cell_B_germline_cyst
28_germline_cell_C_unknown_stage
29_oviduct
30_female_reproductive_system_A
31_female_reproductive_system_B
32_neuron_A
33_neuron_B

D.

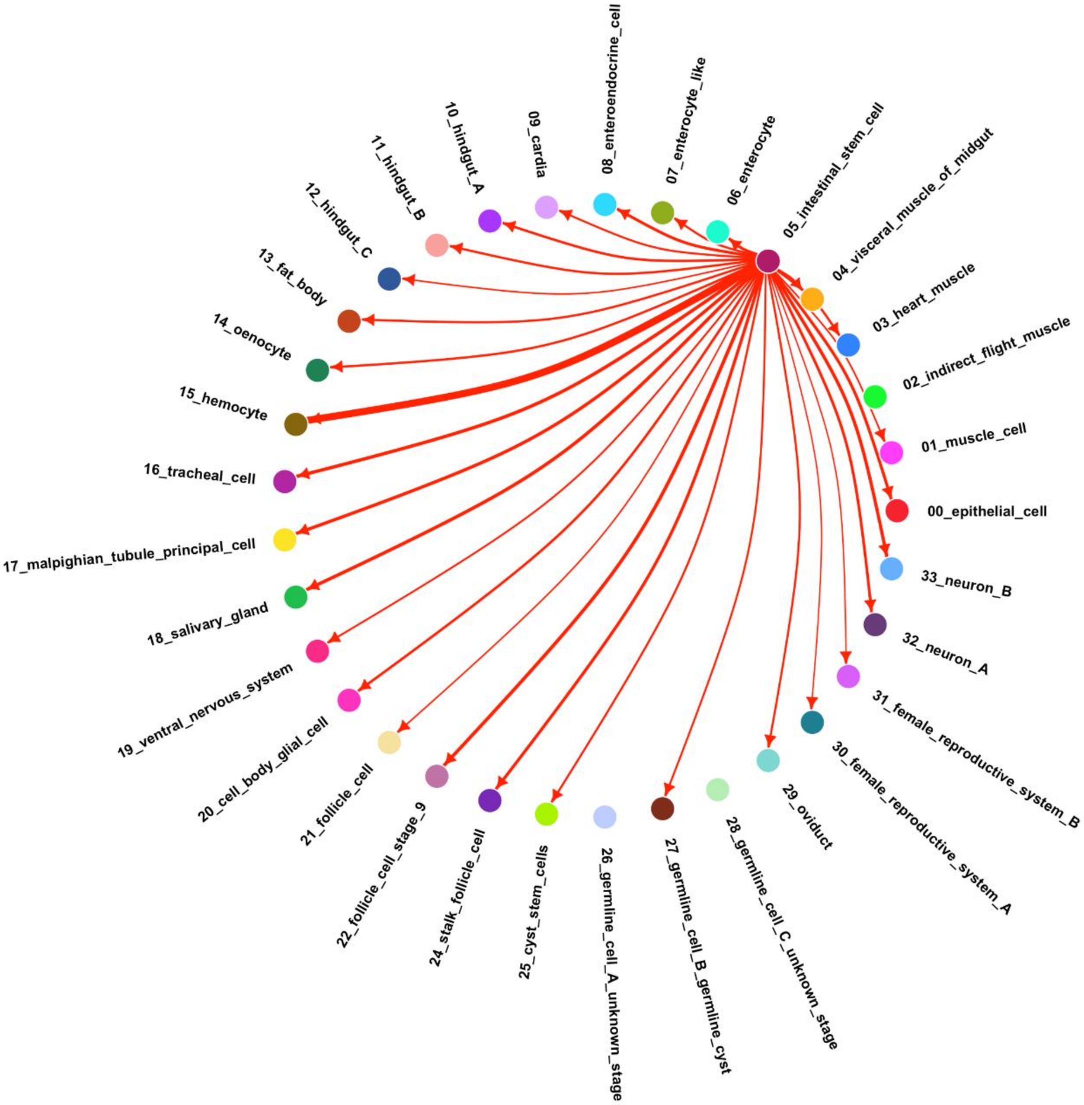

00_epithelial_cell
01_muscle_cell
02_indirect_flight_muscle
03_heart_muscle
04_visceral_muscle_of_midgut
05_intestinal_stem_cell
06_enterocyte
07_enterocyte_like
08_enteroendocrine_cell
09_cardia
10_hindgut_A
11_hindgut_B
12_hindgut_C
13_fat_body
14_oenocyte
15_hemocyte
16_tracheal_cell
17_malpighian_tubule_principal_cell
18_salivary_gland
19_ventral_nervous_system
20_cell_body_glial_cell
21_follicle_cell
22_follicle_cell_stage_9
24_stalk_follicle_cell
25_cyst_stem_cells
26_germline_cell_A_unknown_stage
27_germline_cell_B_germline_cyst
28_germline_cell_C_unknown_stage
29_oviduct
30_female_reproductive_system_A
31_female_reproductive_system_B
32_neuron_A
33_neuron_B
